# Assessment of healthcare workers' knowledge and attitude on Ebola virus disease in Somalia: a multicenter nationwide survey

**DOI:** 10.1186/s12889-023-16562-2

**Published:** 2023-08-28

**Authors:** Abdirahman Khalif Mohamud, Ikran Abdulkadir Ali, Ahmed Isse Ali, Najib Isse Dirie, Pamornsri Inchon, Omar Abdullahi Ahmed, Abdulrahman Ahmed Mohamud

**Affiliations:** 1 Faculty of Medicine and Health Sciences, SIMAD University, Mogadishu, Somalia; 2 Department of Neonatal Intensive Care Unit in Yardimeli Hospital Mogadishu, Mogadishu, Somalia; 3 Dermatology of Department, Mogadishu-Somali Turkey Training and Research Hospital, Mogadishu, Somalia; 4 Department of Urology, Dr Sumait Hospital, Faculty of Medicine and Health Sciences, SIMAD University, Mogadishu, Somalia; 5 Department of Public Health, School of Health Science, Mae Fah Luang University, Chiang Rai, Thailand; 6 Department of ART Unit, Banadir Hospital Mogadishu, Mogadishu, Somalia

**Keywords:** Ebola virus disease, Knowledge, Attitude, Practice, KAP survey, Healthcare workers, Infection prevention and control, East Africa, Somalia

## Abstract

**Background:**

In September 2022, a new Ebola outbreak was reported in Uganda, East Africa, and 142 confirmed cases, including 19 Healthcare workers (HCWs) reported. Ebola is not endemic in Somalia, but the country is at a reasonable risk of the virus being introduced due to the direct connection with daily flights from Uganda without border health control and prevention activities. Therefore, evaluating HCWs' Knowledge and attitude is crucial since this is the first time being evaluated in Somalia. The study's objective is to evaluate the HCWs' Knowledge and attitude toward the Ebola virus disease in Somalia.

**Method:**

An online self-administrated cross-sectional survey was conducted among HCWs (*n* = 1103) in all six federal member states of Somalia using a validated, reliable, well-structured questionnaire. Data we analyzed using descriptive statistics and Logistic regression were used to determine sociodemographic characteristics associated with poor Knowledge and negative attitude.

**Result:**

Over one-third (37.3%) of HCWs had poor Knowledge; the mean knowledge score was 7.97 SD ± 2.15. Almost 40.1% of the HCWs had a negative attitude; the mean attitude was 27.81 SD ± 8.06. Low-income HCWs (AOR = 2.06, 95%CI:1.01–4.19), Married HCWs (AOR = 1.39, 95%CI: 1.110–1.963), Midwives (AOR = 2.76, 95%CI: 1.74–4.39), Lab technicians (AOR = 2.43, 95%CI: 1.43–4.14), HCWs work in Jubaland state of Somalia (AOR = 3.69, 95%CI: 2.39–5.70), Galmudug state (AOR = 8.50, 95%CI: 4.59–15.77), Hirshabelle state (AOR = 3.18, 95%CI: 2.15–4.71) were more likely to have poor Knowledge compared to their counterparts. HCWs who work in Hirshabelle state (AOR = 5.44,95%CI: 3.58–8.27), Jubaland state (AOR = 8.47, 95%CI: 4.69–15.29), and Galmudug state (AOR = 4.43, 95%CI: 3.03–6.48) was more likely to have a negative attitude than those working in the Banadir region administration.

**Conclusion:**

Most Somali healthcare workers showed good Knowledge and a positive attitude toward the Ebola virus. The implementation to enhance Knowledge and attitude must specifically focus on low-income HCWs, Midwives, Lab technicalities, and those who work in Hirshabelle, Jubaland, and Galmudug states of Somalia.

## Background

Ebola virus disease (EVD), also known as Ebola hemorrhagic fever, is a severe and often fatal illness caused by the Ebola virus. The virus is transmitted to humans through contact with infected animals, bodily fluids of infected people, or contact with contaminated objects [1]. After 2 to 21 days of its incubation period, symptoms including fever, headache, muscle pain, weakness, fatigue, diarrhea, vomiting, abdominal pain, and unexplained bleeding or bruising can appear [2, 3]. The Ebola virus is highly contagious, often a feral disease, and licensed treatment and vaccines are available in limited numbers to cure and prevent Zaire Ebola virus species. In contrast, Sudan Ebola virus species (SUDV) currently have no licensed treatment and vaccines [4]. However, supportive care, effective prevention strategies, and infection control practices in healthcare settings are vital to improving the chances of survival [2–5].

WHO recognized a public health threat and numerous vital aspects, including healthcare workers' (HCWs) knowledge and attitude require deep understanding [3–7]. Therefore, enhancing the community's knowledge and attitude is essential to reduce transmission [7–11]. Sub-Saharan African communities over-trust traditional medicine and religious, cultural, and social leaders instead of scientific medical knowledge, which may worsen the outbreaks [12]. Surveying HCWs' knowledge and attitude may help control recurrent outbreaks and support knowledge-building activities [12–14].

Previous Ebola outbreaks caused healthcare workers significant mortalities, and healthcare providers were 21 to 32 times greater to be infected Ebola virus than the general adult population [15]. In Sierra Leone, a high number of healthcare workers were infected Ebola virus, with almost 221 death of healthcare workers [15]. In addition, the outbreak affected numerous efforts to strengthen human resources for health, and a weak healthcare system might exacerbate the situation [16, 17]. Around 500 confirmed cases of healthcare workers were reported in Guinea, Liberia, Sierra Leone, and Nigeria [17]. Liberia, Sierra Leone, and Guinea lost 8%, 7%, and 1% of HCWs, respectively [18, 19]. The outbreak also delayed and adversely impacted healthcare delivery; over 50% occurred in the above three countries [20].

In September 2022, a new Ebola outbreak was reported in Uganda, and 142 confirmed and 22 suspected cases with 77 deaths were reported. Almost 19 confirmed cases with 7 deaths were HCWs [4, 21]. The Ebola outbreak is not currently or previously reported in Somalia, but the country is at risk due to the direct connection with daily flights from Uganda, where the disease is endemic without any border health control and prevention activities. Passenger and military flights from Uganda operate daily in Somalia. The Ministry of Health and its partners reported a reasonable risk of introducing the virus into Somalia and enhanced their emergency response plan [22]. Therefore, these efforts become worthless without evaluating HCWs' knowledge and attitude toward the Ebola virus since it has yet to be surveyed in Somalia. This assessment may have a vital role in developing tailored health education programs, supporting public health policymakers and interventions that focus on implementing response to Ebola and strengthening HCWs' awareness. The study aims to evaluate the HCWs' knowledge and attitude toward the Ebola virus disease in Somalia.

## Methodology

### Study design

A cross-sectional online survey was conducted between 1 December 2022 to 31 January 2023 among healthcare workers across all six federal member states of Somalia. The study invited voluntary, anonymous online surveys to healthcare workers to evaluate KAP toward Ebola.

### Inclusion and exclusion criteria

Healthcare professionals who were directly or indirectly involved in healthcare in all six federal member states, including the Banadir administration, older than 18 years were included in the study after excluding those does not have healthcare qualifications, those who are not Somali citizens, and those who do not provide verbal consent.

### Study population and sampling techniques

**Fig. 1:**
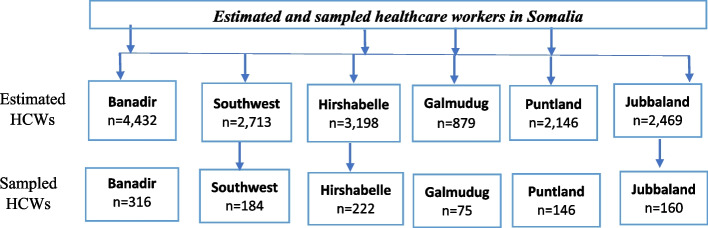
Sample size fraction distribution from Somali federal member state

The healthcare workers in this study were qualified medical professionals directly or indirectly involved in providing healthcare for all six federal member states of Somalia. They included nurses (*n* = 395), medical doctors (*n* = 362), midwives (*n* = 132), Lab technicians (*n* = 89), Pharmacists (*n* = 38), and other healthcare providers (*n* = 87). The Ministry of Health and Social Services of the federal republic of Somalia estimated that 19,306 healthcare professionals work in Somalia [23–25]. Since there was no similar study in the study area, the sample size calculation was based on a single population proportion formula built on the 50% assumption that the probability of having poor knowledge and attitude toward Ebola virus infection with a 3% of margin of error and, 95% confidence interval (CI) [25, 26]. The total sample study required was 1063 healthcare workers including an additional 5% of a non-response rate. However, this study recruited 1103 respondents after excluding 30 incomplete responses. The probability proportional to size (PPS) sampling technique [27] was used to ensure that the sample was represented in each unit and that samples were distributed as below (Fig. 1).

### Development of research tools and variables

A well-structured reliable, validated, self-administered questionnaire was developed from a literature review [8, 13, 28] and discussed by three experts. The questionnaire consists of i) Socio-demographic characteristics (Age, gender, marital status, healthcare profession and experience, monthly income), ii) Twelve true or false simple-dichotomy knowledge statements, and iii) Nine Likert 5-scale attitude statements. All statements are based on Ebola virus infection, epidemiological characteristics, source, signs and symptoms, supportive treatment mode of transmission, risk factors, outbreak prevention, and control strategies.

For the Knowledge section, a 1 score gave a correct response and a 0 for an incorrect response for regular statements and vice-versa for the reverse statements. The maximum expected knowledge score was 12, and all knowledge statements were computed and categorized as poor Knowledge if the score was ≤ 60% (0–7 scores) and good Knowledge if the score was > 60% (8–12 scores) according to the widely adopted modified Bloom's cut-of-point classification [29–32].

For attitude, nine Likert 5-scale statements were used (1 from strongly disagree to 5 strongly agree), and vice versa reverse statements. All statements were computed; the maximum expected score was 45 and was categorized as a positive attitude if the score was > 60% (28 to 45 score) and a negative attitude if the score was ≤ 60% (0 to 27 scores) according to the widely adopted modified Bloom's cut-of-point classification [33, 34].

The study tool was initially developed in English, and then language experts did forward–backward translations to verify the consistency. The Somali version was used to collect data. The item objective congruence (IOC) method [35] was used for content validity by three external experts (An infectious disease specialist, a Tropical medicine physician, and a clinical epidemiology & public health expert). Consequently, to ensure reliability and respondents' understanding of the questionnaire, a pilot study of 55 respondents (5% of the sample) was conducted for respondents with similar characteristics. An acceptable internal consistency Cronbach's Coefficient alpha of 0.83 and 0.81 for Knowledge and attitude statements were achieved, respectively.

### Data collection techniques

The study tool was initially developed in English, and then language experts translated it into Somali to verify the consistency. The Somali version constructed an online electronic form used for data collection. The online tool shared eligible HCWs who met the inclusion criteria and they responded to the self-administered online questionnaire without support. All respondents were informed of the study objective and eligibility criteria, and participation is voluntary and requested to provide verbal consent. The healthcare administrative officials at the healthcare institutions in the six federal member states facilitated the data collection.

### Data analysis

Data were cleaned, coded, entered, and kept on the speeded sheet, then imported into the SPSS version 20 (SPSS, Chicago, IL) for analysis. Descriptive statistics were used for general characteristics and knowledge and attitude statements by presenting frequency with percentage for categorical data and mean with standard deviation (SD) for continuous data. The knowledge section, modified Bloom's cut-of-point classification [29–32], was used by giving 1 score for a correct response and 0 for an incorrect one. All knowledge statements were computed and categorized as poor Knowledge (≤ 60%) and good Knowledge (> 60%). All attitude statements were computed and were categorized as a negative attitude (≤ 60%) and a positive attitude (> 60%) according to modified Bloom's cut-of-point classification [33, 34]. Logistic regression in univariable and multivariable models was used to determine variables associated with poor Knowledge and negative attitude. Variables with a *p*-value < 0.20 in the univariable logistic regression model were candidates for multivariate logistic regression. Bursac et al. [36] suggest that variables with a *p*-value < 0.20 in univariate logistic regression may have some reasonable association with the outcome in the final model if they adjust each other due to the possibility of having a confounder in other variables. The Hosmer–Lemeshow goodness of fit test was used to indicate the final model goodness of fit [37]. In Multivariable logistic regression, variables with a *p*-value < 0.05 were considered statistically significant.

## Result

### Socio-demographic characteristics

A total of 1103 healthcare workers participated in this study; most of them (28.6%) work in the Banadir region and almost half (46.7%) were aged between 26 to 33 years old with a mean 29.25 SD ± 7.85. More than half (54.4%) were female, 35.8% were nurses, 52.0% had 1–5 years of healthcare work experience, and 51.9% had a monthly income between 201–500 USD (Table 1).

**Table 1:** Socio-demographic characteristics

Variables	Frequency	%
**Federal member states**		
Banadir region	316	28.6
Puntland state	146	13.2
Jubaland state	160	14.5
Galmudug state	75	6.8
Hirshabelle state	222	20.1
Southwest state	184	16.7
**Age (years)**		
18 to 25	403	36.5
26 to 33	515	46.7
> 33	185	16.8
Age (years)	Mean ± SD 29.25 ± 7.85	
**Gender**		
Male	503	45.6
Female	600	54.4
**Marital status**		
Unmarried	487	44.2
Ever married	616	55.8
**Profession**		
Nurse	395	35.8
Doctor	362	32.8
Midwife	132	12.0
Lab technician	89	8.1
Pharmacist	38	3.4
Other	87	7.9
**Experience**		
≤ 1 year	217	19.7
1–5 years	574	52.0
6–9 years	220	19.9
≥ 10 years	92	8.3
**Monthly Income USD$**		
≤ 200	208	18.9
201–500	572	51.9
501–1000$	235	21.3
≥ 1,000$	88	8.0

### Healthcare workers' knowledge of Ebola virus infection

This study showed that one-third of 411 (37.3%) healthcare workers had poor knowledge, and 692(62.7%) had good knowledge of Ebola virus infections. The mean knowledge score was 7.97 SD ± 2.15 (Table 2).

**Table 2:** Knowledge of healthcare worker

Level of knowledge	Frequency n(%)	Mean (SD)
Poor Knowledge (≤ 60%) (0–7 scores)	411 (37.3)	7.97 ± 2.15
Good Knowledge (> 60%) (8–12 scores)	692(62.7)	
**Total**	1103(100.0)	
**Knowledge statements**		
**Statement**		**Correct answer n(%)**
This is the first Ebola outbreak^a^		491 (44.5%)
The Ebola virus is an airborne disease, and its transmission occurs through the air		551 (50.0%)
Healthcare workers are at the frontline risk of Ebola disease while treating the patients		932 (84.5%)
Personal protective equipment can prevent infection in the healthcare workers		893 (81.0%)
Sudden onset of fever, intense weakness, and muscle pain are hallmark symptoms		846 (76.7%)
Ebola can be treated easily with only antibiotics^a^		289 (26%)
Touches infected persons and animals, and contaminated body fluids or materials without protection can cause becoming infected		873 (79.1%)
Decreased white blood cell counts and platelet counts are Laboratory findings		862 (78.2%)
Ebola can cause neurological symptoms including temporary paralysis like polio		618 (56.0%)
The incubation period of Ebola is between 2 and 21 days		850 (77.1%)
The Ebola virus can decrease platelet counts and cause severe uncontrollable bleeding		828 (75.1%)
The vulnerable groups are Children and healthcare workers		761 (69.0%)

^a^Reverse statements (False response is the correct answer)

### Healthcare workers' attitude to Ebola virus infection

This study showed that 442 (40.1%) healthcare workers had a negative attitude, while 661(59.9%) had a positive attitude toward Ebola virus infections. The mean attitude was 27.81 SD ± 8.06 mean (Table 3).

**Table 3:** Attitude of healthcare worker

Level of Attitude	Frequency n(%)				Mean ± SD		
Negative attitude (≤ 60%) (0 to 27 scores)	442 (40.1)				27.81 ± 8.06		
Positive attitude(> 60%) (28 to 45 score)	661 (59.9)						
**Total**	1103 (100.0)						
**Attitude statements**							
**Statement**		**Strongly agree n(%)**	**Agree n(%)**	**Undecided n(%)**		**Disagree n(%)**	**Strongly disagree n(%)**
Ebola virus disease (EVD) is a serious illness		463 (42.0)	189 (17.1)	26 (2.4)		127(11.5)	298 (27.0)
The healthcare workers' active participation in hospital infection control programs can reduce EVD prevalence		253 (22.9)	410 (37.2)	68 (6.2)		181 (16.4)	191 (17.3)
Ebola-affected countries should close borders to prevent transmissions		258 (23.4)	305 (27.7)	86 (7.8)		205 (18.6)	249 (22.6)
Infected persons should be isolated and managed separately		416 (37.7)	301 (27.3)	85 (7.7)		145 (13.1)	156 (14.1)
Healthcare workers feel comfortable while providing service to Ebola patients^a^		72 (6.5)	217 (19.7)	84 (7.6)		410 (37.2)	320 (29.0)
Full healthcare facilities preparedness is required to manage the Ebola outbreak		135 (12.2)	401 (36.4)	84 (7.6)		291 (26.4)	192 (17.4)
Following advice regarding the Ebola virus disease can reduce the risk of Ebola		153 (13.9)	479 (43.4)	115 (10.4)		172 (15.6)	184 (16.7)
Washing the dead cases of Ebola virus disease patients without personal protective equipment causes no harm^a^		116 (10.5)	177 (16.0)	67 (6.1)		284 (25.7)	459 (41.6)
Transmission of the Ebola virus can be prevented by practising infection control measures such as complete equipment sterilization		353 (32.0)	373 (33.8)	76 (6.9)		127 (11.5)	174 (15.8)

^a^Reverse statements (5 scores gave strongly disagree to strongly agree)

### Association between demographical characteristics and poor knowledge

In the Univariable logistic regression analytical model, six (6) variables were associated with healthcare workers (HCWs) poor knowledge of Ebola virus infection: These variables were candidates for multivariable logistic regression, and four were associated with poor knowledge. Low-income healthcare workers were 2.06 times more likely to (95% CI;1.01–4.19) have poor knowledge of Ebola virus infections compared to high-income HCWs. The odds of poor knowledge were 1.39 times greater (95% CI; 1.110–1.963) for ever-married HCWs compared to unmarried HCWs. Midwives are 2.76 times more likely (95% CI; 1.74–4.39), and Lab technicians are 2.43 times more likely (95% CI; 1.43–4.14) to have poor knowledge compared to Nurses. Healthcare workers working in Jubaland state were 3.69 times more likely (95% CI; 2.39–5.70), Galmudug state was 8.50 times more likely (95% CI; 4.59–15.77), and Hirshabelle state 3.18 times more likely (95% CI; 2.15–4.71) to be poor knowledge compared to those working Banadir region administration (Table 4).

**Table 4:** Associated between knowledge level and socio-demographic characteristics

Variables	Knowledge level		OR(95%CI)	AOR(95%CI)	*p*-value
	**Poor (%)**	**Good (%)**			
**Federal member states**					
Banadir	86 (27.2)	230 (72.8)	1.00	1.00	
Puntland	16 (11.0)	130 (89.0)	0.32(0.185–0.585)	0.32(0.17–0.60)	< 0.001*
Jubaland	97 (60.6)	63 (39.4)	4.11(2.754–6.158)	3.69(2.39–5.70)	< 0.001*
Galmudug	57 (76.0)	18 (24.0)	8.46(4.718–15.201)	8.50(4.59–15.77)	< 0.001*
Hirshabelle	118 (53.2)	104 (46.8)	3.03(2.113–4.357)	3.18(2.15–4.71)	< 0.001*
Southwest	37 (20.1)	147 (79.9)	0.673(0.435–1.043)	0.64(0.40–1.01)	0.058
**Gender**					
Male	178(35.4%)	325(64.6%)	1.00		
Female	233(38.8%)	367(61.2%)	1.15(0.90–1.48)		
**Age (years)**					
18 to 25	148 (36.7)	255 (63.3)	1.00		
26 to 33	164 (31.8)	351 (68.2)	0.805(0.612–1.059)		
> 33	99 (53.5)	86 (46.5)	1.983(1.394–2.823)		
**Marital status**					
Unmarried	143 (29.4)	344 (70.6)	1.00	1.00	
Ever married	268 (42.5)	348 (56.5)	1.853(1.440–2.383)	1.39(1.03–1.88)	0.032*
**Healthcare profession**					
Nurse	125 (31.6)	270 (68.4)	1.00	1.00	
Doctor	104 (28.7)	258 (71.3)	0.87(0.63–1.18)	0.62(0.42–0.92)	0.018*
Midwife	80 (60.6)	52 (39.4)	3.23(2.20–5.00)	2.76(1.74–4.39)	< 0.001*
Lab technician	49 (55.1)	40 (44.9)	2.64(1.65–4.22)	2.43(1.43–4.14)	0.001*
Pharmacist	22 (57.9)	16 (42.1)	2.97(1.50–5.85)	1.90(0.86–4.22)	0.110
Other	31 (35.6)	56 (64.4)	1.19(0.73–1.94)	1.60(0.92–2.78)	0.091
**Experience**					
< 1 year	70 (32.3)	147 (67.7)	1.00		
1–5 years	239 (41.6)	335 (58.4)	0.98(0.58–1.65)		
6–9 years	72 (32.7)	148 (67.3)	1.47(0.92–2.35)		
≥ 10 years	30 (32.6)	62 (67.4)	1.00(0.59–1.68)		
**Income Monthly US**$					
< 200	38 (43.2)	50 (56.8)	1.40(0.84–2.33)	2.06(1.01–4.19)	0.044*
201–500	232 (40.6)	340 (59.4)	1.26(0.90–1.75)	0.98(0.64–1.48)	0.923
501–1000	68 (28.9)	167 (71.1)	0.75(0.50–1.12)	0.89(0.52–1.52)	0.695
> 1,000	73 (35.1)	135 (64.9)	1.00	1.00	

^*^Significant level at *p*-value < 0.05

### Association between demographical characteristics and negative attitude

In the univariable logistic regression analysis, six (6) variables were associated with negative attitudes: These variables were candidates for multivariable logistic regression, and only one variable was found to be significantly associated with negative attitudes toward Ebola virus infection. Healthcare workers working in Hirshabelle state were 5.44 times (95%CI; 3.58–8.27) more likely to have negative attitudes than those working in Banadir region administration. Those who work in Jubaland state 8.47 times (95%CI; 4.69–15.29) greater of have a negative attitude compared to those who work in Banadir region administration. Those who work in Galmudug state were 4.43 times greater (95%CI; 3.03–6.48) to have a negative attitude compared to those who work in the Banadir region administration (Table 5).

**Table 5:** Associated between attitude level and socio-demographic characteristics

Variables	Attitude level		OR(95%CI)	AOR(95%CI)	*p*-value
	**Negative (%)**	**Positive (%)**			
**Federal member states**					
Banadir	80 (25.3%)	236(74.7%)	1.00	1.00	
Puntland	16(11.0%)	130(89.0%)	0.36(0.20–0.64)	0.39(0.21–0.73)	0.002*
Hirshabelle	135(60.8%)	87(39.2%)	5.33(3.53–8.04)	5.44(3.58–8.27)	< 0.001*
Jubaland	103(64.4%)	57(35.6%)	8.69(4.87–15.51)	8.47(4.69–15.29)	< 0.001*
Galmudug	56(74.7%)	19(25.3%)	4.57(3.16–6.62)	4.43(3.03–6.48)	< 0.001*
Southwest	52(28.3%)	132(71.7%)	1.16(0.77–1.74)	1.13(0.74–1.70)	0.559
**Age (years)**					
18 to 25	166(41.2%)	237(58.8%)	1.00		
26 to 33	181(35.1%)	334(64.9%)	0.77(0.59–1.01)		
> 33	95(51.4%)	90(48.6%)	1.501.06(2.13)		
**Gender**					
Male	194(38.6%)	309(61.4%)	1.00		
Female	248(41.3%)	354(58.7%)	1.12(0.88–1.43)		
**Marital status**					
Unmarried	178(36.6%)	309(63.4%)	1.00		
Ever married	264(42.9%)	352(57.1%)	1.302(1.020–1.662)		
**Profession**					
Nurse	144(36.5%)	251(63.5%)	1.00		
Doctor	124(34.3%)	238(65.7%)	0.90(0.67–1.22)		
Midwife	75(56.8%)	57(43.2%)	2.29(1.53–3.42)		
Lab technician	42(47.2%)	47(52.8%)	1.55(0.98–2.47)		
Pharmacist	34(89.5%)	4(10.5%)	14.81(5.15–42.59)		
Other	23(26.4%)	64(73.6%)	0.62(0.37–1.05)		
**Experience**					
< 1 year	91(41.9%)	126(58.1%)	1.00		
1–5 years	246(42.9%)	328(57.1%)	1.03(0.75–1.42)		
6–9 years	76(34.5%)	144(65.5%)	0.73(0.49–1.07)		
≥ 10 years	29(31.5%)	63(68.5%)	0.63(0.38–1.06)		
**Income Monthly US**$					
< 200	29(33.0%)	59(67.0%)	1.813(1.076–3.053)		
201–500	242(42.3%)	330(57.7%)	1.215(0.883–1.671)		
501–1000	73(31.1%)	162(68.9%)	1.977(1.341–2.914)		
> 1000	98(47.1%)	110(52.9%)	1.00		

^*^Significant level at *p*-value < 0.05

## Discussion

The Ebola virus disease (EVD) outbreak is not currently or previously reported in Somalia. However, the country is at risk due to the direct connection with daily flights from Uganda, where the disease is endemic without meaningful border health control and prevention activities. Passenger and military flights from Uganda operate daily in Somalia [22]. The Ministry of Health (MOH) and its partners reported a reasonable risk of introducing the virus into Somalia. They enhanced their emergency response plan by strengthening capacities following the 2005 international health regulation (IHR) to protect vulnerable populations against emergency health threats. In addition, they strengthened surveillance and alerts systems for any cluster of unexplained fever, bleeding, deaths, or other main EVD clinical features, implemented access to advanced diagnostic laboratories for the Ebola virus, established primary infection prevention and control (IPC) measurements in any health center and provided healthcare workers training to prevent infections from themselves and the community. In addition, a rapid response team was nominated and trained to conduct contact tracing and investigate if suspected case report [22].

Furthermore, in November 2018, the infectious hazard management and control unit of the World Health Organization's Eastern Mediterranean regional office implemented sequenced activities to scale up Ebola preparedness and response due to the risk of importation of cases from the democratic republic of Congo was high where the Ebola outbreak is ongoing at that time. Somalia actively participated in these activities launched by WHO to help these countries prepare for early detection of any possible outbreak, enhancing surveillance capacity, sample collection, and laboratory tests. A rapid response team from Somalia, Libya, and Pakistan received additional training [38]. Similar preparedness efforts helped ensure that all these countries remained free from Ebola between 2014 to 2015 [38]. Before the covid-19 pandemic, Somalia faced numerous man-made and natural disasters and infectious disease outbreaks, including tuberculosis, malaria, measles, viral hepatitis, parasitic infections, diarrhea, and HIV, which is uncommon [39–45].

This study recruited 1103 healthcare workers (HCWs) from Somalia's six federal member states. The study revealed that over one-third of the HCWs lack basic knowledge of Ebola (EVD). In addition, half of HCWs do not know the mode of the disease transmission. In general, poor EVD knowledge is a risk factor; HCWs' poor knowledge has a high impact on the disease spread with severe consequences for the HCWs and the entire community. HCWs with insufficient knowledge may not take the necessary precautions to protect themselves from infection leading to excessive disease spread. A similar study in the Demographic Republic of Congo (DRC) is in line and reported high disease transmission among HCWs, reasoned due to poor transmission knowledge [46].

This study discovered that one-six of the HCWs do not know they are in the frontline risk groups. It may lead HCWs to neglect and not implement infection control practices such as avoiding touching the body of a suspected or confirmed patient or dead body, regular hand washing, using personal protective equipment, and increasing the disease transmission to other healthcare providers and the community. A previous study reported that over 2,127 HCWs confirmed Ebola cases, including 1145 deaths during the EVD outbreak between 2014 and 2015 [16, 17]. It was the worst hazard that African healthcare workers faced [18, 19].

Over a quarter of this study participants do not know the sign and symptoms of the disease and even the incubation period. Hence, only one-quarter knew the mode of disease treatment. HCWs' poor signs and symptoms knowledge may delay disease diagnosis. It might prevent on-time treatment and early action, enhancing the outbreak since the disease is highly contagious and has high mortality rates. A low-income setting like sub–Saharan Africa usually has a healthcare worker shortage that may exacerbate the situation. A similar study is inline [47].

In addition, a similar study in Sub–Saharan Africa reported that previous Ebola outbreaks delayed and adversely impacted healthcare delivery [20]. Healthcare workers' poor knowledge may erode trust in the healthcare system, making outbreak control difficult. Therefore, this study re-emphasizes implementing healthcare educational programs and comprehensive training on EVD, including infection prevention and control, to enhance HCWs' access to up-to-date information and resources to help them provide safe and effective care to themselves and their patients. This study revealed that over 40% of the participating healthcare workers had a negative attitude toward the Ebola virus. Furthermore, many of them cannot believe their active participation in hospital infection control programs can reduce EVD prevalence. That can substantially impact the management and containment of the disease. Healthcare workers' negative attitudes interlink to Ebola patients' stigma and discrimination that discourage patients from seeking medical care and complying with public health measures which ultimately contribute to the spread of the disease and make it more difficult to control. A similar study concluded that healthcare workers' negative attitudes are highly associated with patient stigma. Those with negative attitudes may abuse Ebola patients verbally or/or behaviorally, worsening the situation [48].

In addition, almost one-third of the HCWs do not recognize the importance of following advice regarding the Ebola virus disease can reduce the risk of Ebola. Subsequently, over one-half need to recognize that full healthcare facilities preparedness is required to manage the Ebola outbreak. Poor knowledge can affect HCWs' ability to provide compassionate and effective care to Ebola patients. Without HCWs' preparedness, it might lead to medical treatment errors, compromise the safety of healthcare workers themselves, and may promote treatment misconceptions. Consequently, it can also undermine the healthcare system's trust and make implementing effective public health interventions difficult. Similar studies supported and stated that HCWs' negative attitudes and beliefs lead to ignoring healthcare instruction and addicting inappropriate practices [46, 49]. HCWs' practical training, support, providing needed resources, addressing negative attitudes, and promoting a culture of compassion and respect can become a buffer and valuable solution.

## Conclusion

This study recruited healthcare workers (*n* = 1103) from all six federal member states of Somalia. It concluded that around one-third of the HCWs lack basic knowledge of the Ebola virus disease (EVD), and almost 40% of the HCWs' showed a negative attitude towards the Ebola virus disease (EVD). Therefore, the study re-emphasizes implementing healthcare educational programs and comprehensive training on EVD, including infection prevention and control, to enhance HCWs' access to up-to-date information and resources to help them provide safe and effective care to themselves and their patients. The implementation to enhance knowledge and attitude must specifically focus on low-income HCWs, Midwives, Lab technicalities, and HCWs that work in Hirshabelle, Jubaland, and Galmudug states of Somalia.

## Strengths and limitations of the study

The study has numerous strengths; first, to the best of our knowledge, it is the first similar study assessing healthcare providers' knowledge and attitude toward Ebola virus disease conducted in Somalia; second, it is a multicenter nationwide survey sampled and survived all Somali federal member states (*n* = 1103) so the finding acts as nationally representative data that might help policymakers, healthcare educational programs, and comprehensive training on Ebola. A self-administered questionnaire was used to collect the information, so the respondents' intention and seriousness to the questions responses were difficult to access.

## Data Availability

All data collected and generated during the study are included in this paper.
